# Mucormycosis of the Spine: A Case Report and Review of the Literature

**DOI:** 10.7759/cureus.23623

**Published:** 2022-03-29

**Authors:** Jaimin Patel, Zach Pennington, Andrew M Hersh, Bethany Hung, Daniel M Scuibba, Sheng-Fu L Lo

**Affiliations:** 1 Neurosurgery, Johns Hopkins University School of Medicine, Baltimore, USA; 2 Neurosurgery, Mayo Clinic, Rochester, USA; 3 Neurological Surgery, Northwell Health, New York City, USA

**Keywords:** t-cell all, antifungal, epidural abscess, osteomyelitis, spinal mucormycosis

## Abstract

Mucormycosis is an extremely rare, invasive infection commonly isolated to patients with known immunosuppressed status. In the present case, a 36-year-old woman, with a history of T-cell acute lymphoblastic leukemia in remission, presented with T4 osteomyelitis and an associated epidural collection. Biopsy was consistent with mucormycosis, and the patient was recommended for surgical debridement. After declining debridement, the patient was successfully managed on a multiagent antifungal regimen consisting of intravenous amphotericin B, micafungin, and oral posaconazole. The patient was alive without clear evidence of disease at eight months, representing one of the first cases of spinal mucormycosis infection successfully treated with medical management alone. We additionally review the previous descriptions of spinal mucormycosis infections to identify those interventions most associated with successful clearance or containment of these infections.

## Introduction

Spinal epidural abscesses are an increasingly common clinical pathology, with reports suggesting their presence in approximately one in every 100,000 patients [1] and one in every 10-20,000 hospital admissions in the United States [2,3]. However, the prevalence of this condition can vary based on geographical location. Medical and surgical management paradigms have been described [4,5], with the preferred treatment often depending upon the implicated microbial species. Most infections appear to occur secondary to Streptococcus and Staphylococcus aureus species, including methicillin-resistant S. aureus (MRSA) [4]. However, fungal [6] and mycobacterial infections [7] have been reported. Fungal infections (e.g., Candida and Aspergillus) have been reported to account for <1% of all cases, and they disproportionately affect immunocompromised patients [6]. Rarer still are infections from Rhizopus species (mucormycosis), of which there are only a handful of cases [8-19]. Here, we report a case of spinal mucormycosis in an immunocompromised patient with long-term survival, along with a proposed management algorithm based upon the reported literature.

## Case presentation

A 36-year-old female with a history of T-cell acute lymphoblastic leukemia (T-ALL) presented to the emergency department for management of bilateral lower extremity paralysis and urinary/fecal incontinence. The patient had previously undergone treatment with first-line therapy (prednisone, daunorubicin, vincristine) and second-line therapy (nelarabine and concomitant radiation) for her T-ALL. She was undergoing treatment with intrathecal methotrexate and maintenance methotrexate-vincristine combination therapy when she developed acute onset bilateral lower extremity weakness. Imaging had demonstrated an epidural abscess. A T2-3 laminectomy and epidural fluid drainage were performed at an outside facility, after which she had developed flaccid paralysis of the bilateral lower extremities. Biopsy at this time showed no tumor cells. Of note, her treatment had been previously complicated by a zygomycetes fungal pneumonia nine months prior to presentation, which had been successfully treated with isavuconazole. However, fungal cultures from the epidural fluid were not obtained. Due to her immunosuppressed status, she was on antimicrobial prophylaxis with sulfamethoxazole-trimethoprim, isavuconazole, and acyclovir at the time of admission.

**Figure 1:**
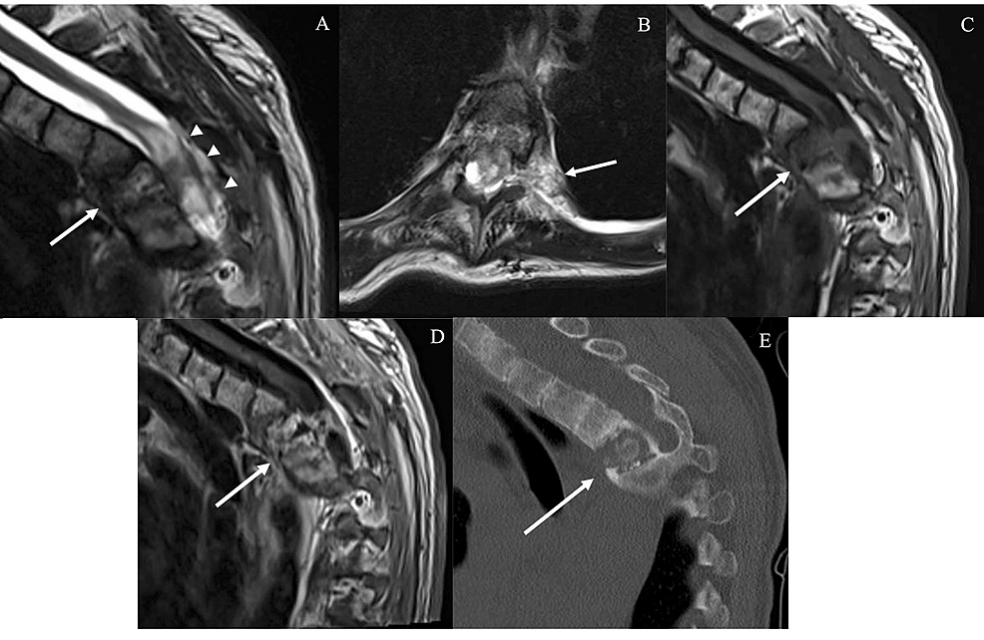
MRI and CT images of the thoracic spine of the patient (A) Sagittal slice from a T2-weighted MRI shows an ill-defined osteodestructive process of the T4 vertebra with anterior wedging (arrow) and enlargement of the posterior T3-5 cord (arrowheads). Also visible on the sagittal slice is the patient’s prior T2-3 laminectomy defect. (B) The axial slice illustrates that the pathology extended from the T4 body into the ventral and left ventrolateral space. There was also a transforaminal excursion into the left pleural space. The presence of a cerebrospinal fluid signal at the T4 level suggested the intrinsic cord T2 signal hyperintensity was likely secondary to ventral compression over the focal T4 kyphosis. (C, D) Sagittal pre- and post-contrast-enhanced T1-weighted FLAIR sequences show heterogeneous enhancement of the mass. (E) A parasagittal non-contrast CT image shows near complete destruction of the T4 vertebra.

On examination, the patient was 0/5 in the bilateral lower extremities, with absent rectal tone and a T6 sensory level. Magnetic resonance imaging (MRI) of the thoracic spine was obtained, which demonstrated collapse of the T4 vertebral body with an enhancing epidural mass circumferentially enveloping the cord and creating a T2-signal cord hyperintensity from the level of T2 to T6 (Figures 1A-1E). The patient was afebrile. Laboratory data demonstrated a leukocyte count of 4.45×10³/μL and a C-reactive protein level of 1.0 mg/dL. β-D-glucan, galactomannan, cryptococcal, and Histoplasma antigen studies were negative. Her urinalysis was found to be positive for extended-spectrum β-lactamase producing Escherichia coli, indicating a potential urinary tract infection of unknown clinical significance.

Because of her known epidural collection lacking microbial characterization and the unrevealing hematology, a biopsy of the epidural collection was performed, which demonstrated heavy hyphal elements on calcofluor white stain, consistent with mucormycosis of the epidural space. Surgical debridement of the bone and drainage of the epidural abscess was recommended; however, the patient declined and opted for medical management. A Hickman catheter was placed, and she was discharged on a multiagent regimen of intravenous amphotericin B (7.5mg/kg daily), intravenous micafungin (100mg daily), and oral posaconazole (300mg daily). After 12 weeks, she was transitioned to monotherapy with oral posaconazole. She remains alive on maintenance posaconazole at eight months follow-up with flaccid paralysis.

## Discussion

Mucormycosis (zygomycosis) is a fungal infection caused by mucormycetes (zygomycetes) molds, most commonly manifesting as cavitary lung lesions or rhinocerebral infection in immunocompromised individuals (e.g., those on immunosuppressive medications) or patients with poorly controlled diabetes mellitus [20]. Microscopy is the gold standard of diagnosis and demonstrates large, nonseptate hyphae branching at right angles with angioinvasion, local tissue necrosis, and neutrophilic inflammation [20,21]. It is capable of spreading rapidly along nerves and blood vessels, leading to invasive mucormycosis, which is known to have a dismal prognosis. Few descriptions of spinal mucormycosis infections exist, and the present case represents only the 11th case in the medical literature (Table 1) [8-19]. This limited literature has precluded the development of optimal management strategies, and to date, there have been no definitive guidelines for managing spinal mucormycosis.

**Table 1:** Literature review of previously published cases of mucormycosis with dissemination to the spine Key: AML – acute myeloid leukemia; ampB – amphotericin B; BID – twice daily; casp – caspofungin; CKD – chronic kidney disease; cx – culture; d – day; DM –diabetes mellitus; F – female; fluC – flucytosine; fu – follow-up; HTN – hypertension; IC – immunocompromised; isov – isovuconazole; itra – itraconazole; IV – intravenous; IVnt –intraventricular; kg – kilogram; L – left; lami – laminectomy; LFU – last follow-up; LN – lymph node; M – male; MDS – myelodysplastic syndrome; mg – milligram; mo – month; n.g. – not given; OM – osteomyelitis; PO – oral; posa – posaconazole; ppx – prophylaxis; R – right; SEA – spinal epidural abscess; s/p – status-post (after); Sx – symptoms; T-ALL – T-cell acute lymphoblastic leukemia; Tx – treatment; vori – voriconazole; wk – week; XRT – radiotherapy

Case	Patient	Species	Location	Immuno-suppressed?	Tx	Outcome
Buruma et al, 1979 [19]	60yo M w/ hx head and neck surgery/ R cervical LN dissection for carcinoma and hx neck XRT + L neck XRT ulcer + tracheostomy Neuro Sx: cervical myelopathy	n.g.	C3-4 OM C1-5 SEA	Y	None	Deceased 1d s/p admission
Rozich 1988 [18]	52yo M w/ hx splenectomy and MDS s/p chemo Neuro Sx: presented with cauda equina syndrome	n.g.	L2-4 SEA	Y	Surg: -L3-5 lami; L4/5 diskectomy -fu L1-2 lami Med: IV AmpB ×12d	Deceased 16d s/p admission
von Pohle 1996 [15]	43yo M w/ DM in diabetic ketoacidosis Neuro Sx: b/l leg weakness→ quadriparesis	n.g.	T3-4 OM; T3-8 meningitis	Y	Med: AmpB @ 1 mg/kg/d ×2d	Deceased 2d s/p admission
Chen et al. 2006 [9]	57yo F w/ recent hx of L4/5 radiofrequency nucleoplasty Neuro Sx: back pain + b/l leg weakness + numbness	R. rhizopodoformis	L4-5 OM + SEA	N	Surg: -L4-5 lami, OM debridement + SEA evacuation -r/p debridement Med: IV AmpB @ 5 mg/d→ 20 mg/d ×8wk +local AmpB wound irrigation @10mg/d ×8wk +PO fluC @ 5 g/d ×3wk +itra @ 200 mg BID ×12mo	Alive w/ disease (small SEA) @ 1yr f/u
Skiada et al. 2009 [14]	2yo M w/ AML on chemo Neuro Sx: status epilepticus + quadriparesis	A. corymbifera	TL junction SEA +intracerebral abscess	Y	Med: Empiric Tx: AmpB @ 5mg/kg/d Post-Cx: PO Vori + PO casp + IVnt AmpB ×1mo Post-PCR: Posa @ 25mg/kg/d +ampB @ 7mg/kg/d ×6mo	Alive w/ disease in vegetative state @ 13mo fu
Tintelnot and Nitsche 2009 [17]	49yo M w/ C6 fracture/sublux 1wk s/p fusion Neuro Sx: None → neck pain + signs of surgical wound infection	R. oligosporus	C/T junction wound infection	N	Med: ampB @ 0.1→ 1mg/kg/d IV ×18d +local H₂O₂ +local povidone-iodine solution +local ampB instillation ×4d	Alive @6mo fu w/ no evidence of disease
Giuliani et al. 2010 [13]	54yo F w/ DM and cutaneous lesion Neuro Sx: T12 sensory level w/ b/l leg paraparesis/paraplegia; long tract signs	R. arrhizus	T10-12 cord lesion	Y	Surg: QD debridement + curettage × ??mo Med: Post Cx: IV ampB @ 300 mg/d ×3mo	Alive at 2yr f/u; unclear disease status
Navanukroh et al. 2014 [12]	42yo F w/ CKD 4d s/p kidney transplant on multiagent immunosuppression Neuro Sx: L leg sciatica	C. bertholletiae	L4-S1 SEA + S1 OM	Y	Surg: L4-S1 lami; 5mL abscess evacuation Med: Empiric Tx: IV AmpB @ 40 mg/d Post-Cx: IV ampB @ 200 mg/d ×3mo + PO posa @ 800 mg/d ×1wk	Alive w/ disease @ LFU
Hadgaonkar et al. 2015 [10]	64yo M w/ hx DM, HTN, CKD Neuro Sx: low back pain; neuro intact	n.g.	L4-5 OM + L4/5 diskitis	Y	Med: IV ampB	Deceased 3wk s/p admission
Shah and Nene, 2017 [8]	54yo M w/ cirrhosis, portal HTN, pancytopenia Neuro Sx: mechanical low back pain + R leg sciatica	n.g.	L3-4 OM	Y	Med: AmpB @ 5mg/kg/d	Deceased 2wk s/p admission
Present Case	36yo F w /hx T-ALL s/p chemo Sx: chronic b/l leg paresis, T6 sensory level	n.g.	T4 OM, T2-6 SEA	Y	Medical: Ppx: PO Isov 372mg/d Tx IV ampB-7.5 mg/kg/d×12 wk, IV micafungin-100 mg/d ×12wk, +PO Posaconazole @ 300mg/d ×12wk Maintenance PO posa @ 300mg/d ×8mo	Alive at 8mo fu; persistent epidural collection at 4mo f/u; no histologic evidence of disease

The first description of spinal mucormycosis was by Buruma et al., who reported an occurrence in a 60-year-old man with a prior history of previously resected oropharyngeal cancer and prior neck irradiation with a persistent radiation-induced neck ulcer [19]. The patient presented with signs of cervical myelopathy, and preliminary imaging showed posterior vertebral body erosions at multiple levels, suspicious for metastatic disease. He rapidly declined, and only on post-mortem were mucor species detected.

In all other reported cases, mucormycosis was diagnosed in time to guide management. However, from these cases, it is apparent that outcomes are extremely poor in immunocompromised patients. Of the eight cases in immunocompromised patients (including the present case), overall mortality has been 50%. Rapid declines were reported in all cases, with von Pohle reporting death two days after admission [15], Rozich reporting death 16 days post-admission [18], Hadgaonkar three weeks post-admission [10], and Shah and Nene documenting death two weeks post-admission [8]. In the three cases besides the present case who were alive at last follow-up, one had persistent epidural disease [12], and another was in a persistent vegetative state secondary to a concurrent intracerebral mucor infection [14]. The only immunocompromised patient to have achieved clearance was a 54-year-old woman treated by Giuliani et al. [13], who had undergone daily debridements and intravenous amphotericin B treatments over months. Unfortunately, she had suffered a T10-12 cord infarction secondary to her angioinvasive mucor disease and was left permanently paraparetic. Nevertheless, this demonstrates the uniqueness of the successful medical management that was achieved in the present patient.

Outcomes for immunocompetent patients have been dramatically better and, as with the Buruma et al. case [19], all patients had a predisposing risk factor for inoculation into the spine. In the first case, the patient had a chronic radiation-induced neck ulcer with underlying carotid artery exposure. This facilitated direct access of cutaneous microbes to the deep tissues of the neck. Similarly, in the case reported by Tintelnot and Nitsche [17], the patient had undergone an open reduction of a C6 fracture-subluxation one week prior to presentation. Likewise, in the case of Chen and colleagues [9], the patient had undergone an L4/5 radiofrequency nucleoplasty shortly before documentation of a mucormycosis infection of the L4 and L5 bodies with ventral epidural expansion.

In all cases, definitive treatment has hinged upon aggressive intravenous antifungal management with amphotericin B. Doses have ranged from 20mg per day to 10mg per kg bodyweight per day, with prolonged courses of 8-12 weeks for patients with long-term reported follow-up. Additionally, in the six patients who have survived at least six months following diagnosis of the infection, rapid surgical debridement was pursued in three [9,12,13]. Those cases in which surgical debridement was not pursued fell into two categories. The first was patients considered to be poor surgical candidates - the present case and that of Skiada et al. [14] - though in our case, surgical debridement was still recommended based upon the poor prognosis without debridement. The second group - the case of Tintelnot and Nitsche [17] was in an immunocompetent adult without evidence of penetration into the vertebral bodies or epidural space. Consequently, evidence at present appears to favor aggressive surgical debridement. In those patients who cannot tolerate surgical debridement, aggressive antifungal regimens are warranted, and the prognosis is likely extremely morbid.

Successful treatment regimens appear to have also included either dual-agent antifungal therapy or local/topical antifungals in addition to intravenous therapy. Four cases, including the present, combined amphotericin with anti-mucormycetes azoles (e.g,, voriconazole, isovuconazole, itraconazole) or echinocandins (e.g., caspofungin, micafungin) [9,12,14]. In two cases, surgical wounds were irrigated daily with colloidal amphotericin B preparations [9,17]. The latter serves to both increase amphotericin concentrations in the infection site, and to allow for the use of lower systemic amphotericin concentrations, as amphotericin is known to be nephrotoxic [9]. This strategy of combined local and intravenous amphotericin B treatment has been described for other invasive mucor infections and may represent an ideal strategy for achieving effective fungicidal levels while minimizing the risk of nephrotoxic injury [22,23]. Lastly, only in the case of Tintelnot and Nitsche was radiographic evidence of disease clearance achieved [17]. This highlights the importance of a long-term antifungal maintenance regimen after completion of the intravenous amphotericin course and remission of all signs of acute infection. Such prophylaxis is especially important in immunocompromised patients and in the present case, oral posaconazole was employed.

## Conclusions

The present case describes the medical management of an invasive mucormycosis infection of the spine in an immunosuppressed patient with a history of T-cell acute lymphoblastic leukemia. Prognosis in such infections is usually poor; however, in those cases that have been documented, successful treatment usually hinges upon aggressive surgical debridement and high-dose intravenous amphotericin B. The addition of local wound irrigation with amphotericin B solutions or dual-agent antifungal regimens including echinocandins or azole antifungals may also help to improve the patient’s likelihood of a good outcome.
